# Missing Numbers

**Published:** 1884-08

**Authors:** 


### MISSING NUMBERS.

The unexpected demand for certain numbers of this journal has resulted in exhausting the edition. We are entirely out of copies of the issues for August, 1883, and for January, 1884. For either of these numbers that may be sent us, we will send a copy of Dr. Miller's "Fermentation in the Human Mouth," or we will pay cash if preferred. Any of our readers who may possess a copy that they have no further use for, will, in addition to receiving the above, confer a great favor upon this journal if they will forward it, at the same time notifying us, so that we may know to whom the obligation is due.

